# The Positivity Rate of IA-2A and ZnT8A in the Chinese Han Population With Type 1 Diabetes Mellitus: Association With rs1143627 and rs1143643 Polymorphisms in the IL1B Gene

**DOI:** 10.3389/fphar.2021.729890

**Published:** 2021-11-11

**Authors:** Jiaqi Li, Xiaoxiao Sun, Shuoming Luo, Jian Lin, Yang Xiao, Haibo Yu, Gan Huang, Xia Li, Zhiguo Xie, Zhiguang Zhou

**Affiliations:** National Clinical Research Center for Metabolic Diseases, Key Laboratory of Diabetes Immunology (Central South University), Ministry of Education, and Department of Metabolism and Endocrinology, The Second Xiangya Hospital of Central South University, Changsha, China

**Keywords:** type 1 diabetes mellitus, single-nucleotide polymorphisms, interleukin 1 beta gene, IL-1β, Chinese han population, association analysis

## Abstract

**Objective:** To investigate the association between susceptibility to type 1 diabetes mellitus (T1DM) and polymorphisms (rs1143627 and rs1143643) in the interleukin 1 beta (IL1B) gene in the Chinese Han population.

**Methods:** The Meso Scale Discovery (MSD) method was used to detect the concentration of IL-1β in 24 T1DM patients and 27 healthy controls. MassARRAY was used to analyze the polymorphisms in the IL1B gene in 510 patients with classic T1DM and 531 healthy controls. The general data of the T1DM patients and healthy controls were compared by the chi-square test and Mann-Whitney U test. The chi-square test and logistic regression were used to analyze the frequency distributions of alleles and genotypes of polymorphisms in the IL1B gene. The Kruskal-Wallis H test and chi-square test were used for the genotype-phenotype analysis of rs1143627 and rs1143643 in the IL1B gene.

**Results:** ① The concentration of IL-1β in T1DM patients was significantly higher than that in healthy controls. ② rs1143627 and rs1143643 in the IL1B gene were significantly correlated with the positivity rates for IA-2A and ZnT8A; genotype GG at rs1143627 and genotype CC at rs1143643 in the case group showed lower positivity rates for IA-2A and ZnT8A. ③ There was no significant difference in the genotypes or allele frequencies at rs1143627 (GG/GA/AA) or rs1143643 (CC/CT/TT) between the case group and control group (*p* > 0.05). ④ rs1143627 and rs1143643 were not found to be linked to T1DM susceptibility under different genetic models.

**Conclusion:** rs1143627 and rs1143643 in the IL1B gene correlate with the positivity rate of IA-2A and ZnT8A in Chinese Han individuals with T1DM**.**

## Highlights

1) T1DM patients had a higher concentration of IL-1β than healthy controls;
2) The rs1143627 and rs1143643 polymorphisms in the IL1B gene are significantly related to the positivity rate of IA-2A and ZnT8A in T1DM patients.

## Introduction

Type 1 diabetes mellitus (T1DM), an organ-specific chronic autoimmune disease, is characterized by insulin deficiency and the resultant hyperglycemia caused by the selective destruction of pancreatic beta cells mediated by T lymphocytes. Genetic and environmental factors contribute to the development and progression of T1DM (DiMeglio et al., 2018; Xie et al., 2018). To date, knowledge of T1DM, including its epidemiology, genetics, immune response, beta cell phenotypes, and disease therapy, has greatly increased. However, the understanding of T1DM pathogenesis is not very clear. Therefore, the pathogenesis and potential directions for future research on T1DM still need to be explored (Skyler, 2018).

T1DM is a disease of polygenic inheritance with strong genetic susceptibility (Pociot and Lernmark, 2016). In addition to the HLA gene family, genome-wide association studies (GWAS) have identified over 60 non-HLA genes related to the risk of T1DM, and these genes participate in the inheritance of T1DM with small genetic effects through their various combinations (Noble, 2015).

Evidence has shown that inflammasome-related genes play an important role in innate immunity (Shaw et al., 2011), and some of them have been found to be involved in the pathogenesis of T1DM under conditions of innate immunity (Van Gorp et al., 2019). IL1B, an inflammasome-related gene, is thought to be the key regulator of both immunity and inflammation. The noticeable and typical characteristic of IL-1β is proteolytic maturation mediated by inflammasomes (Van Gorp et al., 2019). Animal experiments have shown that mitochondrial DNA can activate NLRP3 inflammasomes, which trigger the production of caspase-1-dependent IL-1β and contribute to pathogenic cellular responses in the development of streptozotocin (STZ)-induced T1DM (Carlos et al., 2017). Additionally, IL-1β functions as a driver to boost adhesion molecule expression on immune cells and then acts as a promoter of monocyte tissue infiltration from circulation, thus instigating inflammatory responses (Cai et al., 2020). Elevation of IL-1β levels leads to chronic low-grade inflammation, cytokine profile changes and activation of innate immunity, and the increased expression of IL-1β in plasma/serum can alter lipid metabolism, causing hyperlipidemia (Sonnenschein and Meyle, 2000; Iacopino, 2001). Furthermore, IL-1β may impair insulin release induced by glucose stimulation through a nitric oxide (NO)-independent pathway, unrelated to cell death or glucose metabolism (Andersson et al., 2001). The altered functional state of beta cells is caused by the exposure of human islet beta cells to IL-1β or IL-1β plus IFN-γ; similar findings have been observed in prediabetic patients (Hostens et al., 1999). All of these effects are related to the occurrence of T1DM. Given the known functions of IL-1β in diabetes, we conducted a case-control study to explore the expression levels of IL-1β in T1DM patients.

In recent years, attention has been given to the role of IL1B in the pathogenesis of autoimmune and inflammatory diseases. Numerous studies have demonstrated that there are gene variations and polymorphisms in the IL1B gene and that these variants could influence the transcription and expression of the IL1B gene and are specifically correlated with many autoimmune and inflammatory diseases, including systemic lupus erythematosus (SLE) (Mohammadoo-Khorasani et al., 2016), antisynthetase syndrome (ASSD) (Ponce-Gallegos et al., 2020), rheumatoid arthritis (RA) (Hu et al., 2020) and primary progressive multiple sclerosis (Malhotra et al., 2020). Nevertheless, whether there is a relationship between the IL1B gene and T1DM susceptibility has not been reported before. Therefore, we focused on the Chinese Han T1DM population and selected the IL1B gene as an essential candidate gene. This study aimed to illuminate the correlation between two polymorphisms (rs1143627 and rs1143643) in the IL1B gene and T1DM in the Chinese Han population. Moreover, our study investigated the correlation between IL1B gene polymorphisms and the clinical characteristics of T1DM patients. We hope to present more detailed knowledge concerning the role of polymorphisms in the IL1B gene in T1DM, a specific molecular epidemiological basis for understanding the potential genetic pathogenesis of T1DM, and to provide additional molecular biological indicators for the early diagnosis of this disease.

## Materials and Method

### Participants

The participants were separated into a case group and a control group. Unrelated classic T1DM patients from the Department of Metabolism and Endocrinology at the Second Xiangya Hospital were enrolled in the case group. The selection criteria were as follows: 1) patients meeting the 1999 WHO diagnostic criteria for diabetes; 2) acute onset, and no apparent cause of diabetic ketosis or diabetic ketoacidosis within the previous 6 months; 3) insulin dependence within 6 months of diagnosis; and 4) serum positivity for no less than one of the following islet self-antibodies: glutamic acid decarboxylase antibody (GADA), protein tyrosine phosphatase antibody (IA-2A) and zinc transporter eight antibody (ZnT8A) (Yang et al., 2010). Moreover, the exclusion criteria for the case group were (Xu et al., 2020) 1) secondary diabetes; 2) gestational diabetes mellitus or other special types of diabetes; 3) cooccurrence of other types of autoimmune diseases; and 4) cooccurrence of malignant tumors.

Unrelated healthy volunteers identified through health examinations and epidemiological investigations were enrolled in the control group. The selection criteria were as follows: 1) unrelated individuals of Han nationality residing in Hunan Province; 2) results of a 75 g, 2-h oral glucose tolerance test (OGTT) showing fasting blood glucose (FBG) < 5.6 mmol/L and 2-h postprandial plasma glucose (PPG) < 7.8 mmol/L. The exclusion criteria for this case group were 1) chronic diseases in organs (heart, brain, liver, or kidneys) and/or endocrine disorders; 2) cooccurrence of other types of autoimmune diseases; 3) a family history of diabetes mellitus (DM); and 4) cooccurrence of malignant tumors.

All participants provided signed informed consent, and the ethics committee approved this study of the Second Xiangya Hospital of Central South University.

### Research Methods

#### Collection of Clinical and Biochemical Data

Information collected on all T1DM patients and healthy controls was registered by physicians and included general information, current medical history, diagnosis and treatment process, past and family history, height, weight, waist circumference and hip circumference. Body mass index (BMI), waist-to-hip ratio (WHR), FBG, PPG, triglyceride (TG), cholesterol (TC), high-density lipoprotein (HDL), low-density lipoprotein (LDL) and other biochemical indicators were obtained according to the test requirements. Fasting C-peptide (FCP), postprandial C-peptide (PCP) and glycosylated hemoglobin (HbA1c) were tested by the Endocrine Laboratory of the Second Xiangya Hospital through automated liquid chromatography and chemiluminescence methods. GADA, IA-2A and ZnT8A were detected via radioligand binding assay performed in the Department of Metabolism and Endocrinology, The Second Xiangya Hospital of Central South University.

#### Interleukin 1 Beta Detection

The levels of IL-1β in serum were measured by using the Meso Scale Discovery (MSD) method. MSD detection technology uses SULFO-TAGTM markers. When the electrode surfaces of MULTI-ARRAY and MULTI-SPOT® microplates are energized, the electrochemical action can stimulate SULFO-TAGTM markers to emit strong light. Electrochemiluminescence (ECL) is a specific chemiluminescence reaction initiated by electrochemistry on the surface of the electrode and is a combination of electrochemistry and chemiluminescence. ECL not only has high sensitivity but also can meet the needs of a larger flux. The experiment was completed by the Univ company.

#### Specimen Retention

All subjects signed the informed consent form allowing us to keep their samples. Peripheral blood was collected from each subject and either stored in a freezer at −80°C or used immediately for extraction of genomic DNA. Blood samples were collected from the median cubital vein after an overnight fast and 2 h after a meal. The serum was separated within 2 h, transferred to EP tubes, and stored in a freezer at −80°C.

#### Selection of Candidate Genes and Single-Nucleotide Polymorphisms (SNPs).

The selection of IL1B gene SNPs mainly depends on the loci related to other autoimmune diseases reported in recent years. Moreover, the minor allele frequency (MAF) in the Asian population has to be over 0.05, and the chosen SNPs cannot be located in the same linkage region.

#### Genomic DNA Extraction and Genotyping

The extraction of peripheral blood DNA was performed by the phenol-chloroform method. The DNA samples were sent to BGI (Beijing Genomics Institute, Shenzhen, China) for genotyping of the two polymorphic loci of the IL1B gene, which was performed by mass spectrometry (Agena, MassARRAY® Analyzer 4). The forward and reverse primers for rs1143627 were 5′-ACG​TTG​GAT​GTT​GTG​CCT​CGA​AGA​GGT​TTG-3′ and 5′-ACG​TTG​GAT​GTC​TCA​GCC​TCC​TAC​TTC​TGC-3′, respectively; the forward and reverse primers for rs1143643 were 5′-ACG​TTG​GAT​GAC​TCC​TGA​GTT​GTA​ACT​GGG-3′ and 5′-ACG​TTG​GAT​GCC​TCA​GCA​TTT​GGC​ACT​AAG-3′, respectively.

#### Statistical Analysis

Measurements meeting a normal distribution are expressed as the mean ± standard deviation, and measurements not meeting a normal distribution are presented as the median [interquartile range (IQR)]. Categorical data are expressed as the number of use cases and constituent ratio. The Mann-Whitney U test and chi-square test were utilized to compare the general data between T1DM patients and healthy controls and the differences in categorical data. In the control group, Hardy-Weinberg equilibrium (HWE) was assessed with online software (http://ihg.gsf.de/cgi⁃bin/hw/hwa1.pl) (Sun et al., 2019). Comparisons of allele and genotype frequency distributions between cases and controls were performed using the chi-square test or logistic regression to calculate *p* values, OR values and 95% confidence intervals. The Bonferroni method was used to correct *p* values. The Kruskal-Wallis H test and chi-square test were used for genotype-phenotype analysis of rs1143627 and rs1143643 in the IL1B gene in T1DM patients. The data with a normal distribution were tested by Student’s t-test. All data were analyzed by SPSS 20.0 statistical software (SPSS Inc., Chicago, IL, United States). Statistical significance was defined as *p* < 0.05 for a single test, and a *p* value after correction (pc) < 0.05 was considered statistically significant.

## Results

### The Expression of Interleukin 1 Beta

First, we recruited 24 T1DM patients and 27 age and sex-matched healthy controls. The maximal duration of T1DM was 1 year. All participants were between 11 and 15 years old, and their serum IL-1β levels were measured by the MSD method. The general information, clinical features and serum IL-1β concentration are shown in Table 1. The results showed that there was a difference in HbA1c and TC between T1DM patients and controls and that the HbA1c and TC of T1DM patients were higher than those of the control group (*p* < 0.001, *p* = 0.041). More interestingly, the concentration of IL-1β in the T1DM group was significantly higher than that in the healthy control group (*p* = 0.028). Based on these findings, we further carried out a new study to determine whether IL1B polymorphisms affected T1DM susceptibility in the Chinese Han population.

**TABLE 1:** Descriptive characteristics and IL-1β expression of the T1DM patients and controls.

	T1DM patients	Controls	*p* Value
Sample size	24	27	-
Sex (male/female)	11/13	12/15	0.921
Age (year)	16 ± 4	17 ± 2	0.527
BMI (kg/m^2^)	19.29 ± 2.65	20.54 ± 2.19	0.071
Systolic pressure (mmHg)	111 ± 15	107 ± 13	0.370
Diastolic pressure (mmHg)	70 ± 12	70 ± 10	0.988
HbA1c (%)	9.90 ± 3.33	5.33 ± 0.25	<0.001*
TG (mmol/L)	1.03 ± 0.55	0.91 ± 0.24	0.331
TC (mmol/L)	4.22 ± 0.98	3.71 ± 0.69	0.041*
HDL (mmol/L)	1.43 ± 0.58	1.35 ± 0.21	0.586
LDL (mmol/L)	2.33 ± 1.04	2.06 ± 0.59	0.245
IL-1β (pg/ml)	0.39 ± 0.29	0.23 ± 0.08	0.028*

**Note**: p*<0.05 was considered significant.

Abbreviations:BMI, body mass index; FCP, fasting C-peptide; PCP, 2-h postprandial C-peptide; HbA1c, glycated hemoglobin; TC, total cholesterol; TG: triglyceride; HDL-C, high-density lipoprotein cholesterol; LDL-C, low-density lipoprotein cholesterol.

### Clinical and Biochemical Analysis of Type 1 Diabetes Mellitus and Controls

DNA samples from 510 T1DM patient and 531 healthy controls were included in the analysis. The results regarding age, male-to-female ratio, BMI and sex between T1DM patients and controls have been summarized previously (Sun et al., 2019). No significant difference was found in the sex ratio between T1DM patients and the controls (*p* = 0.418), while the age and BMI of the T1DM group were lower than those of the control group (*p* < 0.01, *p* < 0.01). The FBG and PPG of the T1DM group were significantly higher than those of the healthy control group (*p* < 0.01, *p* < 0.01).

### Comparison of Genotype and Allele Frequencies of the 2 Single-Nucleotide Polymorphisms in Type 1 Diabetes Mellitus Patients and Controls

The 2 SNPs (rs1143627 and rs1143643) in the IL1B gene in this study were in Hardy-Weinberg equilibrium, indicating that the samples were representative of the population (Table 2). The genotypes and allele frequencies of the 2 SNPs are summarized in Table 3. For the rs1143627 polymorphism in the IL1B gene, the distribution frequencies of the 3 genotypes GG, GA and AA in the T1DM group were 20.8, 51.8 and 27.4%, respectively, while those in the healthy control group were 18.1, 52.7 and 29.2%, respectively. The distribution frequencies of allele G in the T1DM group and control group were 46.7 and 44.4%, respectively. The distribution frequencies of allele A in the T1DM group and control group were 53.3 and 55.6%, respectively. There was no significant difference in any genotype or allele frequency between the two groups after adjusting for BMI (*p* > 0.05). For the rs1143643 polymorphism of the IL1B gene, the distribution frequencies of the 3 genotypes CC, CT and TT in the T1DM group were 22.9, 49.5 and 27.6%, respectively, while those in the healthy control group were 21.3, 48.0 and 30.7%. The distribution frequencies of allele C in the T1DM group and control group were 47.6 and 45.3%, respectively. The distribution frequencies of allele T in the T1DM group and control group were 52.4 and 54.7%, respectively. There was no significant difference in any genotype or allele frequency between the two groups after adjusting for BMI (*p* > 0.05).

**TABLE 2:** Hardy-Weinberg Equilibrium of *1LIB* Gene Polymorphisms.

Gene	SNP	Genotype	Observed value	Expected value	χ2	*p* Value
	rs1143627	GG	96	104.89	2.441	0.118
	—	GA	280	262.22	—	—
IL1B	—	AA	155	163.89	—	—
	rs1143643	CC	113	108.93	0.509	0.476
	—	CT	255	263.15	—	—
	—	TT	163	158.93	—	—

**TABLE 3:** Genotype and allele frequencies of rs1143627 and rs1143643 between T1DM patients and controls (numbers of genotypes and alleles (%) or (95% CI)].

Gene	SNP	Cases (N = 510) n (%)	Controls (N = 531) n (%)	*p* Value	OR (95%CI)
	rs1143627	—	—	—	—
	Genotype	—	—	—	—
	GG	106 (20.8)	96 (18.1)	0.476	0.853 (0.550–1.321)
	GA	264 (51.8)	280 (52.7)	0.755	0.947 (0.671–1.336)
	AA	140 (27.4)	155 (29.2)	-	-
	Allele	—	—	—	—
	G	476 (46.7)	472 (44.4)	0.309	1.094 (0.920–1.300)
	A	544 (53.3)	590 (55.6)	-	-
IL1B	rs1143643	—	—	—	—
	Genotype	—	—	—	—
	CC	117 (22.9)	113 (21.3)	0.841	1.039 (0.713–1.514)
	CT	252 (49.5)	255 (48.0)	0.607	1.115 (0.737–1.687)
	TT	141 (27.6)	163 (30.7)	-	-
	Allele	486 (47.6)	481 (45.3)	0.281	1.099 (0.925–1.306)
	C	534 (52.4)	581 (54.7)	-	-
	T	—	—	—	—

OR, odds ratio; CI, confidence interval.

*p* value was obtained by adjusting for BMI.

### Association Between the 2 Single-Nucleotide Polymorphisms and Type 1 Diabetes Mellitus Susceptibility Under Different Genetic Models

Given that the association between susceptibility genes and T1DM is not consistent under different genetic models, we used 4 genetic models to explore the association between IL1B gene polymorphisms and T1DM. The results of this study in the Chinese Han population suggested that there was no risk association between the development of T1DM and the IL1B rs1143627 and rs1143643 polymorphisms in any genetic inheritance model (dominant, recessive, overdominant, and additive) (Table 4).

**TABLE 4:** Genetic models of rs1143627 and rs1143643 between the T1DM group and control group [OR (95% CI)].

SNP	Minor allele	Genetic models							
		Dominant model		Recessive model		Overdominant model		Additive model	
		OR (95% CI)	P	OR (95% CI)	P	OR (95%CI)	P	OR (95%CI)	P
rs1143627	G	1.089 (0.832–1.427)	0.534	1.189 (0.874–1.617)	0.270	0.962 (0.754–1.227)	0.755	1.099 (0.921–1.313)	0.295
rs1143643	C	1.159 (0.887–1.515)	0.279	1.101 (0.822–1.476)	0.519	1.057 (0.829–1.348)	0.654	1.097 (0.925–1.301)	0.286

### Association of the Type 1 Diabetes Mellitus Polymorphisms and Clinical Characteristics

In this study, we further explored whether there is a genotype-phenotype association of the IL1B gene polymorphisms with the clinical characteristics of T1DM patients. We collected peripheral blood from T1DM patients with the GG/GA/AA genotypes of IL1B rs1143627. Analysis of the basic information (sex, age of onset, course of the disease, and BMI), biochemical results (FCP, PCP, HbA1c, TG, TC, HDL, and LDL), and antibody results (GADA positivity rate and titer, IA-2A positivity rate and titer, and ZnT8A positivity rate) of IL1B revealed that T1DM patients with the GG genotype showed a lower rate of positivity for IA-2A and ZnT8A than patients with the GA and AA genotype (*p* < 0.001, *p* = 0.006, Table 5). The ILB gene of rs1143643 was also analyzed in the same way. The results revealed that the CC genotype in T1DM patients was also related to the antibody positivity rate. T1DM patients with the CC genotype displayed a lower rate of positivity for IA-2A and ZnT8A than patients with the CT and TT genotypes (*p* = 0.002, *p* = 0.028, Table 6).

**TABLE 5:** Clinical characteristics of participants with T1DM with different genotypes of rs1143627.

Clinical characteristic	Genotype			*P* Value
	GG	GA	AA	
Sample size	106	264	140	-
Sex (male/female)	56/50	138/126	81/59	0.545
Onset age (year)	20 (10–36)	18 (11–30)	24 (12–32)	0.382
T1DM duration (months)	7.50 (1.00–36.00)	3.00 (0.39–16.00)	6.00 (0.44–14.50)	0.072
BMI (kg/m^2^)	18.66 (16.00–20.80)	18.82 (16.60–20.83)	18.55 (16.44–20.28)	0.541
FCP (pmol/L)	90.00 (16.10–161.90)	66.30 (15.52–165.55)	89.50 (22.40–164.00)	0.380
PCP (pmol/L)	146.03 (26.44–264.70)	126.00 (28.12–273.20)	145.98 (76.26–278.75)	0.376
HbA1c (%)	9.60 (7.35–12.40)	10.20 (7.70–12.70)	10.30 (8.10–13.28)	0.120
GADA positivity (%)	92.40%	86.40%	89.30%	0.247
GADA titer (U/mL)	316.46 (97.48–777.79)	277.34 (84.66–753.34)	315.01 (74.58–804.54)	0.998
IA-2A positivity (%)	28.70%	51.00%	52.30%	<0.00*
IA-2A titer (U/mL)	132.19 (45.36–395.35)	260.19 (56.03–684.39)	123.61 (40.90–696.53)	0.404
ZnT8A positivity (%)	17.00%	35.60%	32.50%	0.006*
TG (mmol/L)	0.94 (0.69–1.51)	0.97 (0.66–1.32)	0.90 (0.73–1.52))	0.763
TC (mmol/L)	4.40 (3.81–6.28)	4.21 (3.53–4.80)	4.30 (3.64–4.85)	0.282
HDL-C (mmol/L)	1.44 (1.10–1.80)	1.22 (1.06–1.61)	1.22 (1.01–1.58)	0.086
LDL-C (mmol/L)	2.30 (1.70–3.19)	2.36 (1.77–2.89)	2.32 (1.76–2.76)	0.923

**Note**: p*<0.05 was considered significant.; **Abbreviations:** GADA, glutamic acid decarboxylase antibody; IA-2A, protein tyrosine phosphatase antibody; ZnT8A, zinc transporter eight antibody.

**TABLE 6:** Clinical characteristics of participants with T1DM with different genotypes of rs1143643.

Clinical characteristic	Genotype			*P* Value
	CC	CT	TT	
Sample size	117	252	141	-
Sex (male/female)	64/53	132/120	79/62	0.771
Onset age (year)	22 (11–35)	17 (11–30)	21 (12–32)	0.370
T1DM duration (months)	6.00 (1.00–24.00)	4.00 (0.48–23.75)	4.00 (0.37–16.00)	0.503
BMI (kg/m^2^)	18.96 (15.97–20.54)	18.71 (16.42–21.00)	18.51 (16.64–20.27)	0.824
FCP (pmol/L)	77.50 (17.50–154.75)	72.60 (14.84–164.10)	79.63 (22.40–167.00)	0.596
PCP (pmol/L)	140.13 (37.28–274.38)	136.67 (27.37–265.88)	140.85 (56.18–277.37)	0.626
HbA1c (%)	9.20 (7.40–12.05)	10.40 (7.90–12.90)	9.90 (8.00–12.80)	0.063
GADA positivity (%)	90.50%	87.30%	88.70%	0.666
GADA titer (U/mL)	312.00 (93.20–714.45)	320.00 (83.21–778.75)	288.75 (79.20–794.11)	0.959
IA-2A positivity (%)	32.40%	49.40%	54.30%	0.002*
IA-2A titer (U/mL)	229.02 (67.50–521.09)	191.02 (64.67–754.91)	156.54 (42.09–640.06)	0.402
ZnT8A positivity (%)	19.80%	34.30%	33.90%	0.028*
TG (mmol/L)	0.89 (0.62–1.28)	1.00 (0.73–1.40)	0.89 (0.65–1.52)	0.482
TC (mmol/L)	4.31 (3.72–4.83)	4.23 (3.63–5.02)	4.34 (3.61–4.85)	0.816
HDL-C (mmol/L)	1.37 (1.01–1.73)	1.22 (1.06–1.59)	1.26 (1.05–1.65)	0.729
LDL-C (mmol/L)	2.36 (1.82–2.92)	2.33 (1.78–2.99)	2.30 (1.72–2.85)	0.966

Note: p* < 0.05 was considered significant.

## Discussion

T1DM is an autoimmune disease in which both genetic and environmental factors influence the disease susceptibility (Xie et al., 2014; DiMeglio et al., 2018). Genetic factors play a critical role in the pathogenesis of T1DM. Many immune-related genetic variants, such as those in HLA genes (Howson et al., 2009) and CTLA4 genes (Ueda et al., 2003), have been confirmed to be related to T1DM. Therefore, attention has been given to specific inherited immune phenotypes that are valid for early T1DM prediction and clinical trials (Todd, 2010).

In addition, T1DM is a T cell-mediated chronic inflammatory autoimmune disease with a strong inflammatory component, which is characterized by specific damage to pancreatic β cells. Innate immunity and inflammatory mediators make significant contributions to the pathogenesis of T1DM. The role of inflammation and its related mediators have an important role in a series of T1DM stages, including insulitis induction, amplification and maintenance or resolution (Eizirik et al., 2009). Additionally, inflammasomes play a vital role in the development and progression of autoimmunity (Shaw et al., 2011), inflammation (Hoffman et al., 2004) and metabolic disease (Vandanmagsar et al., 2011), and many SNPs in inflammasome-associated genes, including NLRP1 (Valdes et al., 2012; Luo et al., 2016; Sun et al., 2019), NLRP3 (Noble et al., 2010), NLRP12 (Borghini et al., 2011) and CARD8 (Noble et al., 2010; Mason et al., 2014), are involved in and associated with patient susceptibility to T1DM.

In our primary study, we first enrolled 24 T1DM patients within 1 year of disease diagnosis and 27 matched healthy controls as a preliminary validation experiment and compared the results against previous studies (Odegaard and Chawla, 2012). The results showed that serum from T1DM patients had a higher concentration of IL-1β than serum from healthy controls, which is consistent with previous studies. Given this result and the current understanding of the IL1B gene in the autoimmune and inflammatory diseases mentioned above, we further explored the association of IL1B gene polymorphisms and T1DM susceptibility.

In addition, the IL1B gene SNPs (rs1143643 and rs1143627) chosen in our study have been demonstrated to be associated with other autoimmune inflammatory diseases, such as RA (Rong et al., 2020), ASSD (Ponce-Gallegos et al., 2020) and inflammatory bowel disease (IBD) (Liu et al., 2020). In view of the potential role of IL1B rs1143627 and rs1143643 in the immune and inflammatory-related diseases mentioned above and the fact that relationships between polymorphisms in the IL1B gene and the risk of T1DM remain discrepant, we aimed to investigate whether the presence of these two polymorphisms (rs1143627 and rs1143643) is associated with T1DM.

This study focused on the Chinese Han population; gene polymorphisms in 510 T1DM patients and 531 healthy controls were analyzed using a case-control study. Their contributions to the genetic risk of T1DM were evaluated. At the same time, we analyzed and compared the differences in clinical data among different genotypes of the IL1B gene. The results showed that both rs1143627 and rs1143643 in the IL1B gene correlated with the antibody positivity rates of IA-2A and ZnT8A. T1DM patients with the CC genotype of rs1143643 showed a lower rate of positivity for IA-2A and ZnT8A than patients with the CT and TT genotypes, and T1DM patients with the GG genotype of rs1143627 also showed a lower rate of positivity for IA-2A and ZnT8A than patients with the GA and AA genotypes. The study did not find that the two candidate SNPs of the IL1B gene are related to susceptibility to T1DM. The limited sample size may be one reason, or it may be that the corresponding genotypes can only delay the progression of T1DM instead of reducing the risk of T1DM.

Our study has the following advantages. It is the first to detect the association between inflammasome-related gene polymorphisms and T1DM in the Chinese Han population. We collected more than 500 cases of T1DM, which is very impressive in the study of T1DM in low-incidence countries. In addition, the sources of our samples are homologous. The DNA samples we used were all from the Han population in Hunan Province, which avoids the influence of genetic heterogeneity in the Han population from different provinces on the accuracy of the results. More importantly, we also analyzed the differences in various clinical characteristics of T1DM patients with different genotypes. However, the findings of this study should be explained in terms of several limitations. First, the number of IL1B gene polymorphisms selected for association analysis was too small. We will consider carrying out a further study with more SNPs and performing a more detailed analysis to explore the impact of gene polymorphisms on the recurrence of T1DM. Second, T1DM is not a monogenic disease but results from multiple gene interactions and interactions between genes and the environment (Todd, 2010). An exploration of the interaction between inflammasome-related gene polymorphisms and HLA susceptibility loci in T1DM was lacking in this study. Finally, to clarify how the specific genetic background leads to the destruction of islet β cells, it is necessary to carry out more extensive and profound functional research on the polymorphisms that have been proven to be associated with susceptibility to T1DM and to further analyze their contribution to T1DM in order to provide a molecular epidemiological basis for a more detailed understanding of the underlying genetic pathogenesis of T1DM and to provide additional molecular biological indicators for the early diagnosis of T1DM.

## Conclusion

Our study found that T1DM patients showed a higher concentration of IL-1β than healthy controls. However, the results did not find an association of rs1143627 and rs1143643 in the IL1B gene with T1DM susceptibility in the Chinese Han population. It was confirmed that both the rs1143627 and rs1143643 polymorphisms in the IL1B gene are significantly related to the positivity rates of IA-2A and ZnT8A in T1DM patients.

## Data Availability

The original contributions presented in the study are included in the article/supplementary files, further inquiries can be directed to the corresponding authors.
